# Longitudinal change in ATN biomarkers in cognitively normal individuals

**DOI:** 10.1186/s13195-022-01069-6

**Published:** 2022-09-03

**Authors:** Jarith L. Ebenau, Denise Visser, Lior A. Kroeze, Mardou S. S. A. van Leeuwenstijn, Argonde C. van Harten, Albert D. Windhorst, Sandeep V. S. Golla, Ronald Boellaard, Philip Scheltens, Frederik Barkhof, Bart N. M. van Berckel, Wiesje M. van der Flier

**Affiliations:** 1 Alzheimer Center Amsterdam, Neurology, Vrije Universiteit Amsterdam, Amsterdam UMC location VUmc, Amsterdam, The Netherlands; 2 Amsterdam Neuroscience, Neurodegeneration, Amsterdam, The Netherlands; 3 Radiology & Nuclear Medicine, Vrije Universiteit Amsterdam, Amsterdam UMC location VUmc, Amsterdam, The Netherlands; 4 UCL Institutes of Neurology and Healthcare Engineering, London, UK; 5 Epidemiology & Data Science, Vrije Universiteit Amsterdam, Amsterdam UMC location VUmc, Amsterdam, The Netherlands

## Abstract

**Background:**

Biomarkers for amyloid, tau, and neurodegeneration (ATN) have predictive value for clinical progression, but it is not clear how individuals move through these stages. We examined changes in ATN profiles over time, and investigated determinants of change in A status, in a sample of cognitively normal individuals presenting with subjective cognitive decline (SCD).

**Methods:**

We included 92 individuals with SCD from the SCIENCe project with [^18^F]florbetapir PET (A) available at two time points (65 ± 8y, 42% female, MMSE 29 ± 1, follow-up 2.5 ± 0.7y). We additionally used [^18^F]flortaucipir PET for T and medial temporal atrophy score on MRI for N. Thirty-nine individuals had complete biomarker data at baseline and follow-up, enabling the construction of ATN profiles at two time points. All underwent extensive neuropsychological assessments (follow-up time 4.9 ± 2.8y, median number of visits *n* = 4). We investigated changes in biomarker status and ATN profiles over time. We assessed which factors predisposed for a change from A− to A+ using logistic regression. We additionally used linear mixed models to assess change from A− to A+, compared to the group that remained A− at follow-up, as predictor for cognitive decline.

**Results:**

At baseline, 62% had normal AD biomarkers (A−T−N− *n* = 24), 5% had non-AD pathologic change (A−T−N+ *n* = 2,) and 33% fell within the Alzheimer’s continuum (A+T−N− *n* = 9, A+T+N− *n* = 3, A+T+N+ *n* = 1). Seventeen subjects (44%) changed to another ATN profile over time. Only 6/17 followed the Alzheimer’s disease sequence of A → T → N, while 11/17 followed a different order (e.g., reverted back to negative biomarker status). APOE ε4 carriership inferred an increased risk of changing from A− to A+ (OR 5.2 (95% CI 1.2–22.8)). Individuals who changed from A− to A+, showed subtly steeper decline on Stroop I (*β* − 0.03 (SE 0.01)) and Stroop III (− 0.03 (0.01)), compared to individuals who remained A−.

**Conclusion:**

We observed considerable variability in the order of ATN biomarkers becoming abnormal. Individuals who became A+ at follow-up showed subtle decline on tests for attention and executive functioning, confirming clinical relevance of amyloid positivity.

## Introduction

The most common cause of dementia is Alzheimer’s disease (AD), which is characterized by the accumulation of amyloid beta plaques and neurofibrillary tau tangles [1]. The ATN classification provides a framework to diagnose AD based on biomarkers providing an indication of these pathologic changes [2]. In this framework, individuals are classified by the presence or absence of amyloid (A), hyperphosphorylated tau (T), and neurodegeneration (N), resulting in eight possible ATN profiles. We previously showed that in cognitively normal individuals with subjective cognitive decline (SCD), A+ was associated with a higher risk of dementia [3].

According to the amyloid cascade hypothesis, the accumulation of amyloid in the brain initiates a series of events including the formation of neurofibrillary tau tangles and neuronal cell loss, eventually resulting in cognitive decline [4]. Therefore, it is assumed that individuals become A+ before turning T+ or N+. Only a few studies evaluated the temporal ordering of ATN biomarker abnormality in a longitudinal manner. One study in a mixed population of cognitively normal individuals and individuals with mild cognitive impairment (MCI) indeed found that most often, A became abnormal first, yet also described there were multiple routes, specifically A → T → N, A → N → T, T → A → N and N → A → T [5].

In the ATN classification, biomarkers are treated as dichotomous variables. With research interest shifting to the very early stages of AD, “grey zone” amyloid burden and subthreshold amyloid accumulation were found to be associated with memory decline, showing additional value of amyloid burden in the perithreshold range [6–8]. One study in a population consisting of cognitively normal and MCI individuals investigated change from A− to A+ rather than accumulation rate and found that lower baseline cognition and APOE ε4 carriership were predictive of changing to A+ [9]. Investigating determinants of amyloid accumulation, specifically in the early stages of disease, therefore has clinical relevance.

The aims of this study were to (1) identify determinants of change in amyloid status, (2) describe changes in ATN profiles over time, and (3) evaluate change in amyloid status as predictor of cognitive decline, in cognitively normal individuals.

## Methods

### Population

We included cognitively normal participants with subjective cognitive decline (SCD) from the Subjective Cognitive Impairment Cohort (SCIENCe) at the Alzheimer Center Amsterdam who had two [^18^F]florbetapir PET scans available (*n* = 92). Individuals were referred to the memory clinic (*n* = 85) by their general physician, a neurologist, or a geriatrician and underwent an extensive standardized diagnostic workup that included a neurologic and neuropsychological examination, laboratory testing, and brain MRI. In a consensus meeting, participants were labeled SCD when cognitive performance appeared within normal limits, and criteria were not met for mild cognitive impairment (MCI), dementia, or other neurological or psychiatric diseases that could possibly cause cognitive complaints. Individuals received a diagnosis of MCI when they had cognitive impairment in one or more cognitive domains, while independence in functional abilities was still preserved [10]. Individuals were diagnosed with dementia when they had cognitive impairment in two or more cognitive domains, which interfered with daily activities [1].

In addition, seven participants were included via the Dutch Brain Research Registry (hersenonderzoek.nl). They experienced cognitive complaints in absence of objective impairment and received the same baseline work-up. At annual follow-up visits, neuropsychological testing was repeated and diagnoses were re-evaluated.

### PET and MRI acquisition

Baseline dynamic [^18^F]florbetapir PET scans were acquired on a Philips Ingenuity TF PET-CT (*n* = 82) or a Philips Gemini TF PET-CT (*n* = 10; Philips, Best, the Netherlands) scanner. These scanners were calibrated to each other. The scan protocol started with a low-dose CT for attenuation correction. Dynamic PET scans of 90 min (*n* = 82) were obtained starting directly after tracer injection of approximately 370 MBq [^18^F]florbetapir. During the course of the study, we demonstrated that scan duration could be reduced without compromising the reliability of results [11]. Therefore, subsequent scans had a duration of 70 minutes (*n* = 9). One scan was terminated early after 79 min due to participant related issues. All underwent a follow-up [^18^F]florbetapir PET with a mean follow-up time of 2.5 ± 0.7 years (*n* = 17 90-min scan; *n* = 75 a 70-min scan). All scans were visually assessed as “positive” or “negative” by a trained nuclear physician, blinded to the amyloid status at the other time point.

Baseline dynamic [^18^F]flortaucipir PET scans were acquired on a Philips Ingenuity TF PET-CT scanner (Philips, Best, the Netherlands, *n* = 44). Because substantial tau pathology within A− cognitively normal individuals is not expected to be present, we selected more A+ individuals for the [^18^F]flortaucipir PET in order to have a broader spectrum of amyloid and tau pathology, resulting in an A+ rate of this subset of about 33%. The scan protocol started with a low-dose CT for attenuation correction. Starting simultaneously with tracer injection of approximately 240 MBq [^18^F]flortaucipir, a 60-min dynamic emission scan was initiated. After a 20-min break and following a second low-dose CT for attenuation correction, an additional dynamic emission scan was performed during the interval 80–130 min post-injection. This dual time point protocol was validated previously [12]. Forty-two individuals underwent a follow-up [^18^F]flortaucipir PET scan using the same procedure with a mean follow-up time of 2.1 ± 0.3 years.

Baseline structural MRI images were obtained at five different systems (GE Discovery MR750 3T (*n* = 22), Philips PETMR 3T (*n* = 51), Signa 1.5T (*n* = 1), Titan 3T (*n* = 17), and external scan (*n* = 1)). The protocol included 3D T1-weighted images, 3D T2-weighted images, and 3D T2-weighted fluid-attenuated inversion-recovery (FLAIR) images [13]. T1-weighted images were used for coregistration to PET images and for determination of the N status. Follow-up MRI was available for 79 individuals with a mean follow-up time of 2.9 ± 0.9 years.

### Image analysis

Data were reconstructed while using standard LOR RAMLA reconstruction algorithm with corrections for dead time, decay, attenuation, random coincidences, and scatter. Images were reconstructed with a matrix size of 128 × 128 × 90 and a voxel size of 2 × 2 × 2 mm^3^. For [^18^F]flortaucipir, both scan sessions (0–60 and 80–130 min) were co-registered into a single dataset of 29 frames (1 × 15, 3 × 5, 3 × 10, 4 × 60, 2 × 150, 2 × 300, 4 × 600, and 10 × 300 s), in which the last 10 frames belonged to the second PET session. 3D T1-weighted MR images were co-registered to PET images using Vinci software (Max Planck Institute, Cologne, Germany). Next, regions of interest (ROIs) were defined on the co-registered MRI using the probabilistic Hammers brain atlas [14] in PVElab. Receptor parametric mapping (RPM) was used to generate parametric binding potential (BP_ND_) images with cerebellar grey matter as a reference region using PPET [11, 15–17]. For [^18^F]florbetapir, we calculated (volume weighted) mean cortical BP_ND_ in a composite ROI consisting of orbitofrontal, temporal, parietal, anterior cingulate, posterior cingulate, and precuneus regions [6, 18].

### Biomarkers: a, T, N

Availability of biomarker status at two time points differed for each of the biomarkers (A: *n* = 92; T: *n* = 42; N: *n* = 79). For 39 individuals, a complete ATN profile over time could be constructed. The time difference with the [^18^F]florbetapir scan was 0.05 ± 0.15y for [^18^F]flortaucipir scans and 0.16 ± 0.62y for MRI scans. We used visual assessment of [^18^F]florbetapir PET scans to define A in the ATN classification. Since quantitative threshold-based methods usually have a high degree of concordance with visual assessment [19, 20], we chose visual assessment to be consistent with methods used in clinical practice. In additional analyses, we used continuous mean cortical BP_ND_ in a composite ROI. We used [^18^F]flortaucipir PET scans as biomarker for T. We pragmatically used Gaussian mixture modeling as an unbiased, data-driven approach, to obtain a threshold. We first averaged values for the anterior part of the lateral temporal lobe for left and right sides. Since the focus of this study was on cognitively unimpaired individuals, we a priori decided to select this region of interest to capture the earliest changes in neocortical areas [21]. We then fit Gaussian Mixture Models with two components using the normalmixEM function in R. A threshold was derived representing the mean of the calculated mu of both components, resulting in a threshold 0.08 BP_ND_. This threshold separated the two clusters with minimal overlap. We used the average medial temporal atrophy rating (MTA) on MRI as biomarker for N as determined by experienced neuroradiologists. Raters were blinded to amyloid status. For individuals < 65 years of age, an average MTA score of ≥ 1 was considered positive; for individuals ≥ 65 years of age, an average MTA score ≥ 1.5 was considered positive [22]. Additionally, white matter hyperintensities were visually assessed using the Fazekas scale (range 0–3) [23]. Microbleeds were assessed on T2-weighted images and defined as small dot-like hypointense lesions. They were counted and dichotomized into absent (0) or present (≥ 1 microbleed).

### Neuropsychological tests

All participants underwent annual standardized neuropsychological assessments [13]. For the memory domain, we used the Visual Association Test version A (VAT-A) and the total immediate and delayed recall condition of the Dutch version of the Rey auditory verbal learning task (RAVLT). For the language domain, we used category fluency (animals). For the attention domain, we used the Trail Making Test A (TMT-A) and Stroop task I and II (naming and color naming). For the domain of executive functioning, we used the TMT-B and Stroop task III (color-word). For global cognition, we used the Mini Mental State Examination (MMSE). Because the data were right-skewed, the raw test scores for TMT and Stroop were log transformed. Subsequently, values were inverted, so that a lower score implies worse test performance for all tests. We used available test results of visits before as well as after PET scans, in order to accurately estimate the cognitive slope. The neuropsychological tests administered most closely to baseline [^18^F]florbetapir were defined as baseline test results. In total, we used longitudinal cognitive data covering 4.9 ± 2.8 years. The proportion of missing tests ranged from 2.7% for MMSE to 8.5% for Stroop II and III. In total, 447 neuropsychological investigations of 92 patients were available (92 ≥ 2 visits, median 4).

### Statistics

All analyses are conducted in R version 4.0.3. We first compared demographic and clinical variables between baseline A− and A+ individuals using *t*-test, chi-square, or Mann-Whitney *U* test where appropriate.

Next, we described changes in biomarker status over time. We first investigated changes in A, T, and N biomarkers separately and then combined in ATN profiles. Then, we examined changes in amyloid status more closely. We categorized (change in) amyloid status as a four-level variable: negative at baseline and follow-up (*negative-negative*), negative at baseline and positive at follow-up (*negative-positive*), positive at baseline and negative at follow-up (*positive-negative*), and positive at baseline and follow-up (*positive-positive*). We investigated change in amyloid status in relation to actual BP_ND_ values at baseline and follow-up, using a division into low, grey zone, and high BP_ND_ with previously described thresholds by our group of 0.19 and 0.29 BP_ND_ [6]. Next, we investigated which factors were associated with change from a negative to a positive amyloid status using logistic regression analyses. In model 1, baseline age, sex, education, baseline MMSE score, and APOE ε4 carriership were evaluated as individual predictors, with the group remaining A− at follow-up as reference group. In model 2, all predictors were entered simultaneously. In an additional analysis, we used amyloid accumulation rate as outcome, using linear mixed models (outcome: BP_ND_ composite ROI). We again assessed baseline age, sex, education, baseline MMSE score, and APOE ε4 carriership as predictors. In model 1, variables were assessed individually, and each analysis included the variable of interest, time, and the interaction between the variable and time. Model 2 included all predictors simultaneously (including time and all interactions between predictors and time).

Last, we used change in amyloid status as predictor of cognitive test performance over time, using linear mixed models. We used our four-level variable reflecting change in amyloid status, time, and their interaction as predictors (*negative-negative* (reference), *negative-positive*, *positive-negative*, and *positive-positive*). Baseline age, sex, and education were used as covariates. Outcome were neuropsychological test scores. Models included a random intercept and additionally a random slope when it improved the model (random slope included for VAT-A, TMT-A, Stroop I-III and MMSE). Separate analyses were run with different tests as outcome.

## Results

### Demographics

Ninety-two individuals were on average 65 ± 8 years old, 39 (42%) were female and 27 (31%) APOE ε4 carrier (Table 1). At baseline, 24 (26%) individuals were A+. By design, A+ individuals had higher baseline amyloid burden. Additionally, they were more often APOE ε4 carrier and had higher baseline tau burden than those who were A−. MTA score, Fazekas score, number of microbleeds, and baseline neuropsychological test scores did not differ between A− and A+ individuals. There were no significant differences in baseline demographics between individuals with complete ATN biomarker information at follow-up and those who did not have complete ATN biomarker information at follow-up available.

**Table 1:** Baseline demographics

	*n*	Total (*n* = 92)	Baseline amyloid negative (*n* = 68)	Baseline amyloid positive (*n* = 24)
Age^a^	92	65.29 ± 8.02	64.82 ± 7.81	66.61 ± 8.61
Sex, *n* female (%)^b^	92	39 (42%)	29 (43%)	10 (42%)
Education, median [IQR]^c^	92	6 [5–6]	6 [5–6]	6 [5–7]
APOE ε4 carriers, *n* (%)^b^	87	27 (31%)	14 (21%)	13 (62%)*
[^18^F]florbetapir BP_ND_^c^	92	0.16 ± 0.12	0.11 ± 0.05	0.30 ± 0.16*
[^18^F]flortaucipir BP_ND_^c^	44	0.01 ± 0.08	− 0.01 ± 0.05	0.05 ± 0.11*
MTA score^c^	92	0.25 [0–1]	0.50 [0–1]	0.00 [0–1]
Fazekas^c^	91	0.96 ± 0.82	0.96 ± 0.84	0.96 ± 0.75
Microbleeds, *n* (%)^b^	92	21 (23%)	15 (22%)	6 (25%)
VAT-A^c^	92	11.61 ± 0.94	11.62 ± 0.98	11.58 ± 0.83
RAVLT immediate^a^	92	44.64 ± 7.68	44.76 ± 7.07	44.29 ± 9.37
RAVLT delayed^a^	92	9.00 ± 3.26	9.34 ± 3.02	8.03 ± 3.75
Animal fluency^c^	92	24.30 ± 6.22	24.09 ± 6.16	24.92 ± 6.49
TMT-A^c^	92	34.89 ± 11.85	34.43 ± 12.11	36.21 ± 11.24
TMT-B^c^	92	81.03 ± 40.55	80.93 ± 44.89	81.33 ± 25.21
Stroop I^c^	91	43.85 ± 9.06	44.18 ± 10.12	42.92 ± 5.13
Stroop II^c^	90	59.48 ± 11.64	60.13 ± 12.49	57.57 ± 8.68
Stroop III^c^	90	94.64 ± 22.33	95.61 ± 22.91	91.83 ± 20.79
MMSE^c^	92	28.86 ± 1.14	28.93 ± 1.15	28.67 ± 1.13

Baseline demographics for the total sample and for amyloid negative and positive individuals separately. Data is presented as mean ± SD unless otherwise specified. Baseline amyloid status is determined by visual read of [^18^F]florbetapir PET. [^18^F]florbetapir BP_ND_ is calculated in a composite ROI. [^18^F]flortaucipir BP_ND_ is calculated in lateral temporal gyrus. MTA score is calculated by averaging right and left sides. Number of microbleeds is dichotomized into 0 counts and ≥ 1 counts; *n* shown is the number of participants with ≥ 1 count. Neuropsychological test scores shown represent baseline values at the visit closest to the baseline [^18^F]florbetapir scan

*BP*_*ND*_ binding potential, *MTA* medial temporal atrophy, *VAT* visual association test, *RAVLT* Rey auditory verbal learning task, *TMT* trail making test, *MMSE* mini mental state examination. ^a^*t*-test; ^b^chi-square test; ^c^Mann-Whitney *U* test. *p < 0.05

### ATN biomarkers over time

**Fig. 1:**
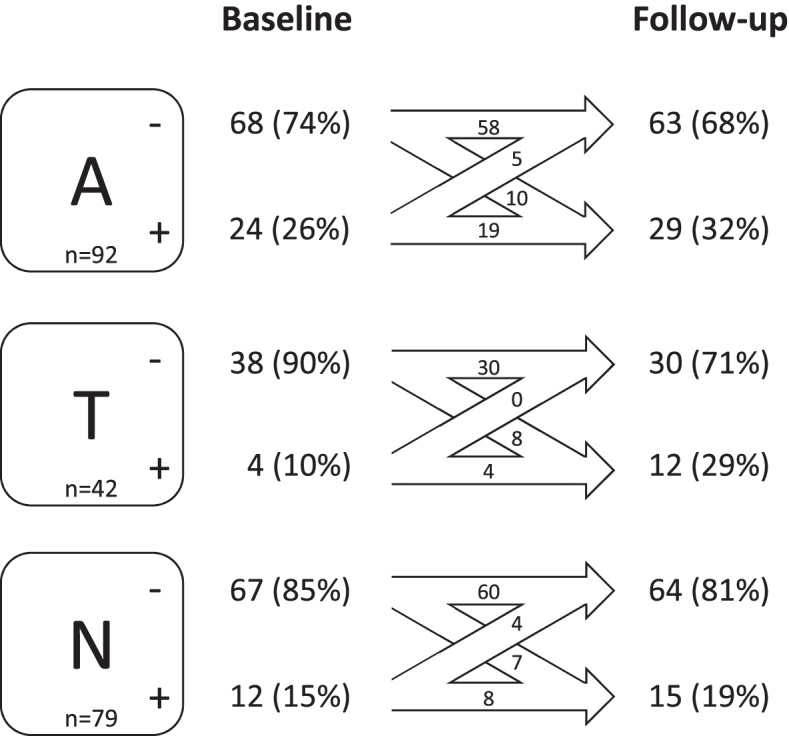
Changes in biomarker status for A, T, and N biomarker groups. Visualization of longitudinal changes in biomarker status for A, T, and N biomarker groups

We first examined changes in A, T, and N biomarkers individually. Figure 1 shows changes in biomarker status over time. Several individuals changed from negative to positive status (A: *n* = 10 (11%); T: *n* = 6 (14%); N: *n* = 7 (9%)). For A and N but not for T, a smaller number of individuals changed from a positive to a negative status (A: *n* = 5 (5%); N: *n* = 4 (5%)).

**Fig. 2:**
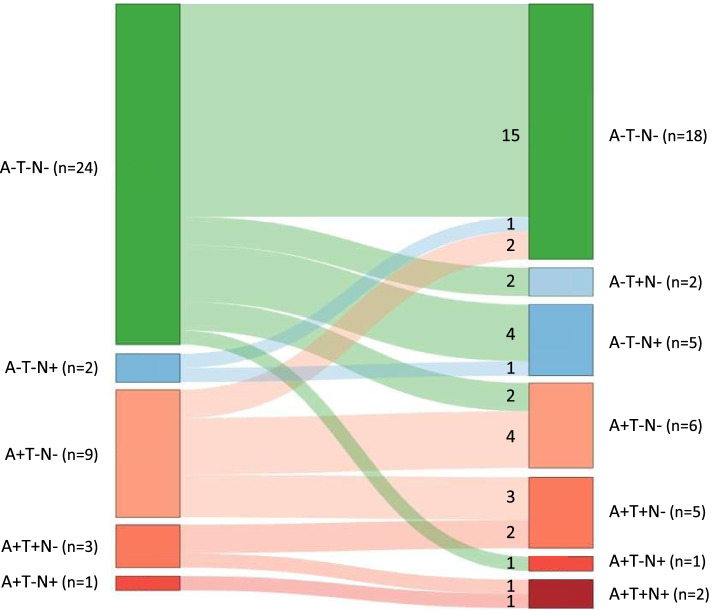
Changes in ATN profiles. Sankey diagram showing changes in distribution of ATN profiles at baseline and follow-up. REM, rapid eye movement; MCI, mild cognitive impairment; AD, Alzheimer’s disease

For individuals with complete biomarker information for A, T, and N at two time points (*n* = 39), Fig. 2 visualizes the trajectory of ATN profiles from baseline to follow-up. During follow-up, 17 (44%) changed to another ATN profile. The percentage of individuals with normal AD biomarkers changed from 62% at baseline to 46% at follow-up. Five percent of individuals fell within the category of non-AD pathologic change at baseline, which changed to 18% at follow-up. The percentage of individuals with biomarkers in the Alzheimer’s continuum changed from 33% at baseline to 36% at follow-up.

According to the most common model of the pathophysiology of AD, the expected sequence of biomarkers becoming abnormal would be A → T → N. Only six of the 17 individuals changing profile fit into this hypothetical sequence. The other eight changed to T+ or N+ while still being A−, or changed to N+ before T+. Finally, three participants changed to a better ATN profile (A−T−N+ → A−T−N− *n* = 1, A+T−N− → A−T−N− *n* = 2).

During the period of follow-up, three individuals showed clinical progression; to dementia with Lewy bodies (DLB) *n* = 1, MCI due to AD *n* = 1, and AD dementia *n* = 1. The individual who progressed to DLB changed from A−T−N− to A+T−N−.The individual who progressed to MCI due to AD had the A+T−N− profile at baseline and at follow-up. The individual who progressed to AD dementia changed from baseline A+T+N− to A+T+N+. The last two individuals both carried an APOE ε4 allele.

### Change in amyloid status over time

**Fig. 3:**
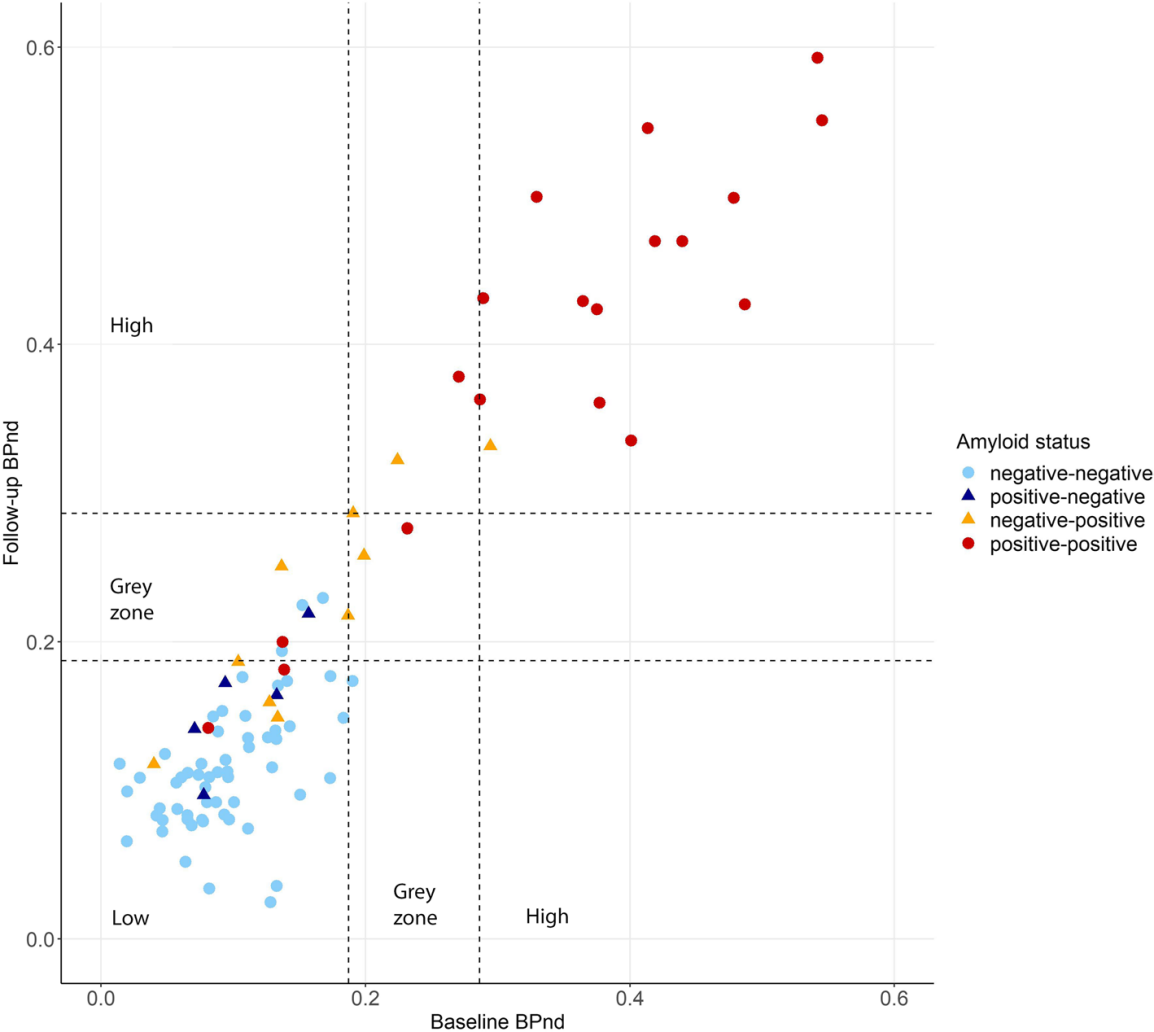
Changes in amyloid status. Scatterplot showing baseline and follow-up BP_ND_ values. Different colors represent individuals with a negative amyloid PET scan at baseline and follow-up (negative-negative), a positive scan at baseline and a negative scan at follow-up (positive-negative), a negative scan at baseline and a positive scan at follow-up (negative-positive), and a positive scan at baseline and follow-up (positive-positive), respectively. The dashed lines represent a division in low, grey zone, and high amyloid burden and is based on a previous study by our group [6], with thresholds of 0.19 and 0.29 BP_ND_. BP_ND_, binding potential

To evaluate amyloid accumulation over time, Fig. 3 visualizes changes in amyloid status in relation to actual BP_ND_ values at baseline and follow-up, using a division into low, grey zone, and high BP_ND_. Overall, BP_ND_ in the composite ROI increased with 0.011 (SE 0.002) annually (*p*-value 0.00). Most individuals who were negative at baseline and at follow-up on visual rating (i.e., *negative-negative*) had low baseline and low follow-up BP_ND_ (54/58), and most *positive-positive* individuals had high baseline and high follow-up BP_ND_ (14/19). The amyloid status of 15 individuals changed over time (*positive-negative n* = 5, *negative-positive n* = 10). Most *positive-negative* individuals had both low baseline and low follow-up BP_ND_ (4/5). The group of *negative-positive* individuals was quite heterogeneous in terms of BP_ND_ values and had at baseline and follow-up low (4/10), grey zone (1/10), or high (1/10) BP_ND_. Two changed from low baseline BP_ND_ to grey zone BP_ND_ at follow-up, and two changed from grey zone BP_ND_ at baseline to high BP_ND_ at follow-up. When conversely focusing on the six individuals with grey zone BP_ND_ at baseline, three belonged to the *negative-positive* group, two to the *positive-positive* group, and one to the *negative-negative* group. This shows a considerable part of this group is on the verge of transitioning to a visually positive amyloid status.

**Fig. 4:**
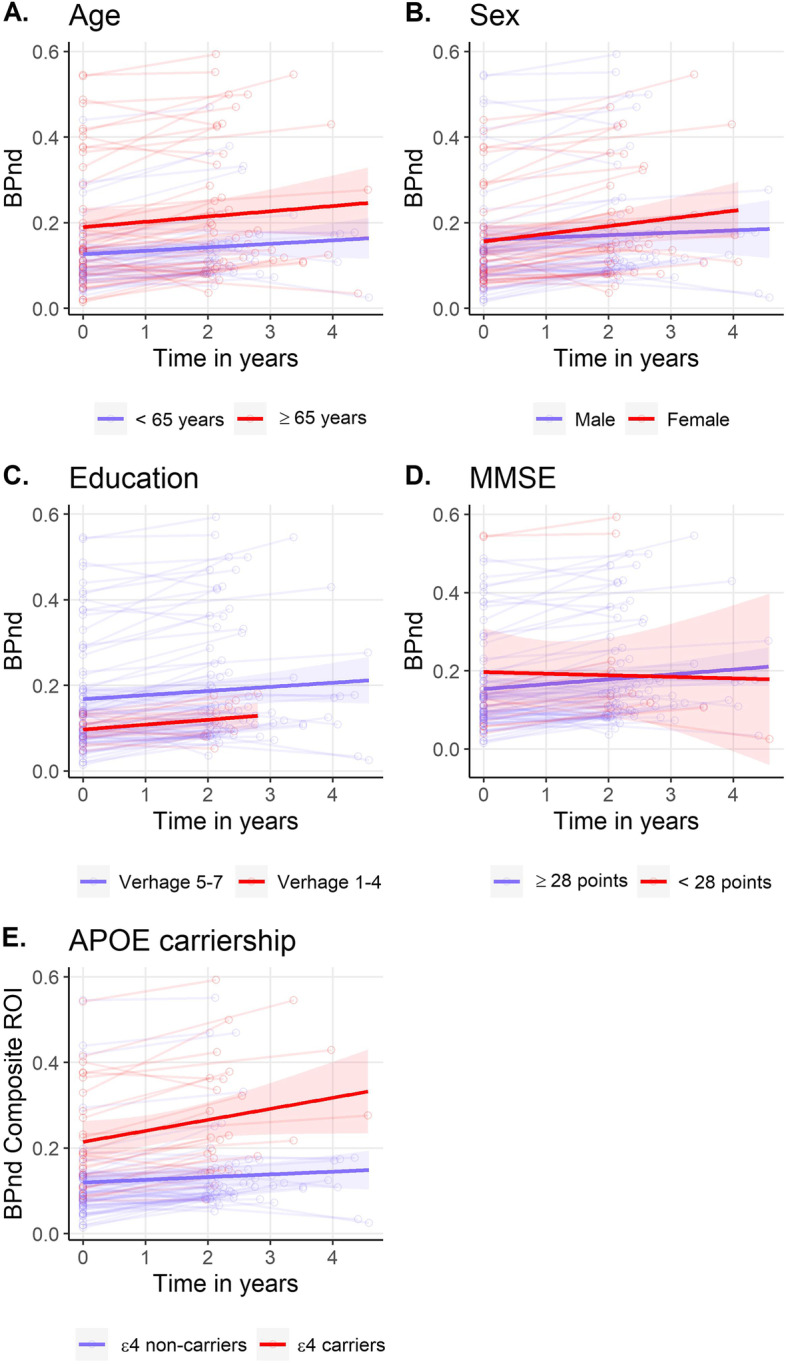
Longitudinal trajectory of amyloid burden. Longitudinal trajectory of [^18^F]florbetapir BP_ND_ over time. Separate lines represent the trajectories for different values of age (**A**), sex (**B**), education (**C**), baseline MMSE (**D**), and APOE ε4 carriership (**E**) respectively

Subsequently, we investigated which factors predicted a change in amyloid status from negative to positive. Logistic regression analysis showed that APOE ε4 carriers had higher odds of transitioning to a positive amyloid status (OR 5.22 (95% CI 1.23–22.75), Table 2). Baseline age, sex, education, and baseline MMSE score were not associated with a higher odds of changing to amyloid positivity. When we analyzed amyloid accumulation rate as continuous outcome using linear mixed models (BP_ND_; Table 3, Fig. 4), we confirmed that APOE ε4 carriers had both higher baseline BP_ND_ values and a higher accumulation rate.

**Table 2:** Change from negative to positive amyloid status

	Model 1	Model 2
Age	0.97 (0.88–1.06)	0.99 (0.88–1.09)
Sex	0.88 (0.21–3.41)	0.45 (0.07–2.16)
Education	0.90 (0.47–1.85)	0.84 (0.37–1.96)
Baseline MMSE	1.50 (0.79–3.45)	1.40 (0.70–3.42)
APOE ε4 carrier	5.22 (1.23–22.75)*	6.39 (1.26–38.41)*

Data shown are odds ratio (95% confidence interval) as estimated by logistic regression. Outcome was conversion from a negative to a positive amyloid status, as compared to remaining amyloid negative. In model 1, age, sex, education, baseline MMSE, and APOE ε4 carriership were investigated as predictors individually. In model 2, all variables were included simultaneously. *p < 0.01

**Table 3:** Associations with baseline amyloid burden and amyloid accumulation rate

	Baseline		Longitudinal	
	Model 1	Model 2	Model 1	Model 2
Age	0.02 (0.01)	0.00 (0.01)	0.00 (0.00)	0.00 (0.00)
Sex	0.04 (0.22)	− 0.04 (0.20)	0.05 (0.03)	0.02 (0.03)
Education	0.23 (0.11)*	0.15 (0.10)	0.00 (0.02)	− 0.00 (0.02)
Baseline MMSE	− 0.05 (0.10)	− 0.09 (0.08)	0.01 (0.01)	0.01 (0.01)
APOE ε4 carrier	0.83 (0.20)*	0.85 (0.21)*	0.11 (0.03)*	0.10 (0.03)*

Data shown are beta (SE) as estimated by linear mixed models. Outcome was [^18^F]florbetapir over time in a composite region of interest. Models included the variable of interest, time, and their interaction as predictors. In model 1, each variable was investigated as predictor individually. In model 2, all variables were included simultaneously. Baseline estimates represent the association between the predictor and baseline BP_ND_, longitudinal estimates represent the association of the interaction between predictor and time and reflect the slope of BP_ND_. * *p* <0.05

### Associations between changes in amyloid status and cognitive test performance

Finally, we investigated whether change in amyloid burden was associated with cognitive test performance over time, using our four-level variable (*negative-negative* (reference), *positive-negative*, *negative-positive*, *positive-positive*) as determinant. We did not find associations between change in amyloid status and baseline cognitive test performance (Table 4). By contrast, we found several associations with cognitive slope, as individuals in the *negative-positive* group had a steeper slope than *negative-negative* individuals on Stroop I and III. *Positive-positive* individuals showed a steeper decline on RAVLT immediate and delayed recall, TMT-B, and Stroop III. *Positive-negative* individuals did not differ from *negative-negative* individuals with regards to their cognitive test performance.

**Table 4:** Associations between change in amyloid burden and cognitive test performance

	Baseline			Longitudinal		
	Positive-negative	Negative-positive	Positive-positive	Positive-negative	Negative-positive	Positive-positive
VAT-A	0.20 (0.30)	0.16 (0.21)	0.05 (0.16)	0.01 (0.11)	0.03 (0.05)	0.01 (0.04)
RAVLT immediate	3.24 (3.36)	0.65 (2.40)	− 2.28 (1.88)	− 0.11 (0.88)	0.04 (0.36)	− 1.40 (0.32)*
RAVLT delayed	0.88 (1.18)	0.55 (0.85)	− 0.96 (0.66)	− 0.03 (0.30)	0.05 (0.12)	− 0.51 (0.10)*
Animal fluency	3.48 (2.13)	1.42 (1.51)	0.79 (1.18)	0.71 (0.57)	− 0.05 (0.23)	− 0.23 (0.21)
TMT-A	0.01 (0.11)	0.13 (0.08)	0.05 (0.06)	0.03 (0.04)	− 0.04 (0.02)	− 0.01 (0.02)
TMT-B	0.04 (0.13)	0.10 (0.10)	− 0.07 (0.08)	0.01 (0.03)	− 0.02 (0.01)	− 0.02 (0.01)*
Stroop I	0.05 (0.07)	0.06 (0.05)	0.01 (0.04)	0.01 (0.02)	− 0.03 (0.01)*	− 0.01 (0.01)
Stroop II	0.06 (0.07)	0.00 (0.05)	0.04 (0.04)	0.00 (0.02)	− 0.02 (0.01)	− 0.02 (0.01)
Stroop III	0.12 (0.09)	0.01 (0.06)	0.02 (0.05)	− 0.01 (0.02)	− 0.03 (0.01)*	− 0.02 (0.01)*
MMSE	− 0.23 (0.48)	0.06 (0.34)	− 0.29 (0.26)	− 0.14 (0.18)	0.01 (0.11)	− 0.06 (0.08)

Data is presented as beta (SE) as estimated by linear mixed models. Models included a four level variable indicating change in amyloid status (*negative-negative* (reference), *negative-positive*, *positive-negative* and *positive-positive*), time, and their interaction as predictors. All models were adjusted for age, sex, and education. Outcome was cognitive test performance. Baseline estimates represent the association between the predictor and baseline test performance; longitudinal estimates represent the association of the interaction between predictor and time and reflect the slope of cognitive test performance. *VAT* visual association test, *RAVLT* Rey auditory verbal learning task, *TMT* trail making test, *MMSE* mini mental state examination. *p < 0.05

## Discussion

In our sample of cognitively normal individuals with SCD, we found that biomarker abnormality increased over a 2.5-year period. There was considerable variability in the order of biomarkers becoming abnormal in the ATN classification, suggesting no fixed order. Change from A− to A+ was associated with steeper decline in tests of attention and executive function.

We showed that the number of individuals with an abnormal biomarker status increased for both A, T, and N, over a time course of 2.5 years. Most of these individuals changed from a negative to positive biomarker status, yet of note, a smaller number of individuals changed from positive to negative. There are not many longitudinal studies investigating changes in the ATN classification; hence, the phenomenon of change from a positive to a negative status has not yet received much attention. One study investigating amyloid burden excluded individuals who were amyloid positive at one time point, and negative at the next, but did not specify the number of individuals [9]. Another excluded the 5% of individuals with borderline amyloid PET burden, reducing the risk of individuals crossing the threshold due to small changes [5]. Other studies investigating amyloid accumulation rate as continuous measure showed a negative slope in some individuals but did not address the possibility of reverting amyloid status and explained the negative slope by random variation, noise, or actual clearance of amyloid [7, 8]. One potential explanation for the five individuals changing from a positive to negative amyloid status by visual rating *(positive-negative)* could be that these scans were false-positive at baseline. While the quantitative measures might not have changed much over time, scan could be visually assessed differently at the two time points due to an imperfect intra-rater agreement [24–26]. This is part of clinical practice, especially in early disease stages. Therefore, acquiring follow-up scans is highly useful in individuals with equivocal scans with grey zone amyloid burden. Of note, one could also argue that also the *negative-positive* scans could be the result of rater variability and that their changing from negative to positive does not necessarily reflect clinical relevance. However, a substantial portion of these individuals had grey zone amyloid burden at baseline, which is already associated with a changed slope in memory function, as shown by our group previously [6]. We found that this group has a steeper decline in performance on Stroop I and III, which are indicative of attention and executive functioning. This is in line with other studies showing that amyloid burden in the subthreshold range is associated with cognitive decline and highlights the clinical relevance of grey zone amyloid burden [7, 8]. In general, an amyloid status based on visual assessment is not identical to an amyloid status based on a threshold for quantitative measures. Quantitative measures are not directly affected by rater variability and could therefore be interpreted as more consistent. However, quantitative measures of amyloid burden are often averaged over a larger ROI. If a scan is visually assessed as A+ based on a relatively small area, this does not necessarily translate in a higher average BP_ND_ in the total ROI, which could be a potential cause of differences between the two approaches. Overall, we found a relatively high degree of changing biomarkers in a short time frame. These results add to the literature suggesting the clinical relevance of changing from a negative to a positive amyloid status.

When we compared ATN profiles over time, we found 44% of individuals changed to a different ATN profile during 2.5 years of follow-up. Data on changing biomarkers enable the evaluation of the actual sequence of biomarker abnormality. Of note, most (11/17) individuals followed a different sequence than the overall accepted hypothesis of A becoming abnormal first, then T and N last [2]. In our sample, individuals changed to T+ or N+ while still being A− or changed to N+ before T+. These findings are in line with those of a former study investigating change in ATN profiles, which also found multiple sequences [5]. There are several possible explanations for these observations. First, amyloid could already be accumulating in the subthreshold range in individuals changing to T+ or N+, but before A+, suggesting the pathological process has started just below the detection threshold. An alternative explanation is the suggestion of the dual-pathway hypothesis, in which amyloid and tau accumulation are both the result of a common upstream event, not necessarily causally related to each other [27]. Finally, there could be mixed pathology, resulting in N+ due to other diseases than AD, hence not related to a specific ordering of events. Overall, the number of individuals with the A−T−N− profile became smaller and the number of individuals with non-AD pathologic change (A−T+N−, A−T−N+, A−T+N+) became larger at follow-up. In a previous study by our group, but also in other studies, these profiles did not have a higher risk of cognitive decline or clinical progression to MCI or dementia [3, 28].

When we evaluated determinants of change to amyloid positivity, we found APOE ε4 carriers had a higher baseline amyloid burden, a higher risk of transition from A− to A+ and a higher annual amyloid accumulation rate. Several studies confirm a relationship between ε4 carriership and a higher accumulation rate [29–31], although not all [7, 32]. The relationship between ε4 carriership and a higher risk of change from A− to A+ has also been confirmed [9, 33]. We add to these results with the finding that ε4 carriership is also associated with risk of change in a sample of cognitively normal individuals with SCD. We did not find evidence for an association with any of the other factors examined, such as baseline age, sex, or education level. In apparent contrast with former studies [9, 30], we did not find a relationship between a lower baseline cognitive performance and subsequent amyloid accumulation. Reasons for this inconsistency could be that an inclusion criterion for our study is normal performance at baseline and that variability in baseline cognition is small. Therefore, relationships with amyloid accumulation may be obscured. In short, our results suggest A− individuals who are ε4 carrier are still at risk of progression to A+.

Limitations of our study include that our sample size was relatively small. With a larger sample size, our study would have had more power to detect more subtle determinants of changes in A status. The results of our analyses examining changing amyloid status as predictor of cognitive decline should also be interpreted with caution and replicated in larger samples. Furthermore, we used [^18^F]flortaucipir PET as measure of tau burden. We pragmatically used Gaussian mixture modeling of [^18^F]flortaucipir to obtain a threshold, although there might be other approaches. Nevertheless, our approach resulted in a percentage of T+ which lies within the range of T+ described in other studies in cognitively normal individuals [3, 34–36]. Of note, during the recruitment of individuals for the [^18^F]flortaucipir PET scan, we slightly oversampled A+ individuals. Because substantial tau pathology within A− cognitively normal individuals is not expected to be present, we selected more A+ individuals for the [^18^F]flortaucipir PET in order to have a broader spectrum of amyloid and tau pathology. Therefore, our results might not reflect the true prevalence of amyloid and tau pathology in cognitively normal individuals and the results might not be directly generalizable to the general population. Another factor that potentially impacts generalizability is the fact that the individuals in our sample were mainly recruited at a memory clinic. Strengths include the longitudinal nature of the study with the availability of biomarkers, diagnoses, and cognition with substantial duration of follow-up. Furthermore, we used dynamic scan protocols which enabled us to calculate BP_ND_, which is a more accurate measure of amyloid and tau load than the semi-quantitative SUVr. Another strength is our use of [^18^F]flortaucipir for the definition of “T,” since it does not suffer from off-target binding to amyloid plaques or TDP-43 and correlates well with Braak neurofibrillary tangle stages [37].

Concluding, we showed biomarker status changes in cognitively normal individuals with SCD. There was considerable variability in the sequence of ATN biomarkers becoming abnormal, suggesting that there is not one (causal) order of events. Changing from a negative to positive amyloid status was associated with APOE ε4 carriership and predicted subtle cognitive decline, suggesting the potential clinical relevance of amyloid burden in the negative range.

## Data Availability

The datasets supporting the conclusions of this article are available upon reasonable request.
